# Prospecting of Novel Angiotensin I-Converting Enzyme Inhibitory Peptides from Bone Collagen of *Pelodiscus sinensis* by Computer-Aided Screening, Molecular Docking, and Network Pharmacology

**DOI:** 10.3390/foods15040663

**Published:** 2026-02-12

**Authors:** Jiaxin Chen, Ruoyu Xie, Yimeng Mei, Wenxuan Chen, Jun Hu, Haoyu Liu, Hongying Du, Guijie Hao, Xiaolong Ji, Shuangxi Li, Jin Zhang

**Affiliations:** 1 College of Food and Bioengineering, Zhengzhou University of Light Industry, Zhengzhou 450001, China; 15737159661@163.com; 2 Zhejiang Key Laboratory of Intelligent Food Logistic and Processing, State Key Laboratory for Quality and Safety of Agro-Products, Institute of Food Science, Zhejiang Academy of Agricultural Sciences, Hangzhou 310021, China; 13127410550@163.com (R.X.); 18532399615@163.com (Y.M.); chenwx@zaas.ac.cn (W.C.); hujun@zaas.ac.cn (J.H.); 15275539718@163.com (H.L.); 3 Department of Food Science and Engineering, College of Light Industry and Food Engineering, Nanjing Forestry University, Nanjing 210037, China; hydu@njfu.edu.cn; 4 Key Laboratory of Healthy Freshwater Aquaculture, Ministry of Agriculture and Rural Affairs, Key Laboratory of Fish Health and Nutrition of Zhejiang Province, Huzhou Key Laboratory of Aquatic Product Quality Improvement and Processing Technology, Zhejiang Institute of Freshwater Fisheries, Huzhou 313001, China; wangfeiyan0804@163.com; 5 Xingzhi College, Zhejiang Normal University, Lanxi 321100, China

**Keywords:** softshell turtle, collagen, ACE-inhibitory peptide, molecular docking, network pharmacology, in silico screening

## Abstract

Hypertension is a globally prevalent chronic cardiovascular disease, with angiotensin-converting enzyme (ACE) serving as a key target for therapeutic intervention. Synthetic ACE inhibitors have side effects, making natural food-derived ACE-inhibitory peptides a research hotspot owing to safety advantages. Softshell turtle (*Pelodiscus sinensis*) bone collagen (STBC) has potential bioactivity, but its ACE-inhibitory peptides have not been systematically investigated. This study used computer-aided screening: STBC α1(I) (K7FHL1) and α2(I) (K7G8R1) sequences from UniProt were processed via SignalP 5.0. BIOPEP-UWM analysis showed ACE-inhibitory peptide frequencies of 0.8947 and 0.9261 in the two chains, confirming STBC as a high-quality precursor. Papain-ficin was selected as the optimal enzymatic system via simulation; 105 potential novel peptides were obtained after toxicity/allergenicity prediction. Twenty-seven highly active peptide fragments were screened out via pLM4ACE, and four peptide fragments with relatively high binding energy (QICVCDS, DVWK, IIEY, APMDVG) were identified through molecular docking. These peptides (molecular weight: 536.6–766.9 Da) possessed excellent physicochemical properties and pharmacokinetic characteristics, while bioinformatics analysis revealed that they could target and regulate SRC/HSP90AA1 to modulate the renin-angiotensin system (RAS). This study provides an efficient strategy for the high-value utilization of softshell turtle resources and the development of food-derived ACE-inhibitory peptides.

## 1. Introduction

Hypertension ranks among the most widespread chronic cardiovascular diseases globally, representing a formidable challenge to international public health. According to statistics from the World Health Organization (WHO), roughly 1.4 billion adults between the ages of 30 and 79 suffered from hypertension in 2024, a figure corresponding to around 33% of this demographic, yet fewer than one-fifth (320 million individuals) had their condition adequately controlled. Moreover, hypertension and its related complications continue to be counted among the primary contributors to premature death on a global scale [1]. Hypertension involves a complex pathogenesis, with the renin-angiotensin system (RAS) playing a central mediating role in the onset and progression of the disease. As a key enzyme within this regulatory system, angiotensin-converting enzyme (ACE) catalyzes the conversion of angiotensin I to angiotensin II, which functions as a strong vasoconstrictor, and concurrently degrades bradykinin along with other vasodilatory peptides. Therefore, inhibiting the activity of ACE has been widely recognized as an effective strategy for treating and preventing hypertension [2,3,4].

Currently available synthetic ACE inhibitors, such as lisinopril, exhibit significant efficacy in clinical applications. However, their side effects (fatigue, hypotension, renal dysfunction, and cough), long-term tolerance, and safety concerns have prompted researchers to focus on natural ACE-inhibitory peptides as potentially safer and less toxic alternatives [5,6]. Food-derived antihypertensive peptides have emerged as a highly promising research focus, owing to their advantages of high safety, minimal side effects, and suitability for long-term administration [6]. Currently, a variety of peptides with ACE-inhibitory activity have been isolated from food sources such as animal, marine, and plant sources. Isolating these potent bioactive peptides from diverse food sources represents a promising approach for dietary management in nutraceuticals and hypertension prevention [7].

Softshell turtle (*Pelodiscus sinensis*) is an economically important aquatic species native to Asian regions, including China, Korea, Russia (Far East), and Japan. China ranks as the world’s top producer of softshell turtles, with an annual yield exceeding 500,000 metric tons [8]. Since ancient times, soft-shelled turtles have been widely recognized as both an edible food and a medicinal material due to their abundant nutritional value. Particularly in the field of traditional medicine, they are considered to have inherent characteristics that contribute to extending lifespan. According to existing reports, softshell turtle peptides possess the efficacy of reducing bodily fatigue and oxidative stress, thereby promoting health and extending lifespan [9,10]. It is estimated that collagen constitutes around 30% of the total protein content in animals and is broadly distributed across diverse tissues and organs, where it performs a pivotal function in sustaining their structural stability and physiological functions. Collagen peptides not only possess ACE inhibitory activity but also exhibit high bioavailability [8,11,12]. Moreover, softshelled turtle calcified tissues, such as its bones, are recognized as containing collagen with the highest concentration and purity, and thus may serve as an excellent source of collagen for pharmaceutical and cosmetic applications [13]. Based on the known biological properties of collagen peptides, we hypothesize that STBC may contain a variety of novel bioactive peptides with potent ACE-inhibitory activity; these peptides can bind to the active site of ACE through specific molecular interactions, thereby inhibiting ACE activity and regulating the RAS. However, no systematic studies have yet identified ACE-inhibitory peptides from softshell turtle collagen, and the potential of such peptides to modulate the RAS and their underlying structural and functional characteristics remains entirely unexplored. Additionally, softshell turtle bones, a valuable TCM component of softshell turtles, have not been investigated as a source of collagen-derived ACE inhibitory peptides, representing a notable research gap in the development of natural hypotensive peptides from aquatic medicinal resources.

Traditional methods for identifying ACE-inhibitory peptides from protein hydrolysates require extensive enzymatic digestion, purification, peptide characterisation, and activity assays, proving laborious and time-consuming. In contrast, computational approaches integrate protein databases, enzymatic digestion simulations, molecular docking, and network pharmacology to rapidly predict a peptide’s inhibitory capacity against ACE alongside its intrinsic properties. This strategy effectively screens bioactive sequences while reducing experimental costs [14,15].

Therefore, this study will adopt computer-aided technologies to explore and identify novel and highly efficient ACE inhibitory peptides that may exist in the bone collagen of soft-shelled turtles. Furthermore, molecular docking and network pharmacology will be employed to conduct a multi-dimensional analysis of these novel peptides for elucidating their structure-activity relationships. This research will facilitate the development and utilization of aquatic animals such as soft-shelled turtles for the integration of food and medicine, and meanwhile provide a new strategy for the computer-aided mining of ACE inhibitory peptides.

## 2. Materials and Methods

### 2.1. Acquisition and Processing of Softshell Turtle Protein Sequences

Among all known types of collagen, type I collagen ranks first in terms of distribution scope and demonstrates exceptionally varied practical applications, particularly as it makes up 90–95% of the organic content in animal bones. Type I collagen and its corresponding derived peptides have been widely applied in the food, cosmetic, and pharmaceutical fields, exhibiting diverse biological activities and considerable economic benefits [16]. This protein possesses a triple-helix supramolecular structure and consists of two α1(I) chains and one α2(I) chain [17]. To obtain two turtle bone collagen sequences (α1(I) and α2(I)) from the UniProt Knowledgebase database (https://www.UniProt.org/, accessed on 11 June 2025), the species and protein name, *Pelodiscus sinensis* collagen, was first entered into the search box. Subsequently, the α1(I) and α2(I) protein sequences were selected according to the detailed protein classification and the relative levels of Annotation score, with the assigned accession numbers of the finally obtained two turtle bone collagen sequences (α1(I) and α2(I)) being K7FHL1 and K7G8R1, respectively. Subsequently, the deep neural network analysis server SignalP 5.0 (https://services.healthtech.dtu.dk/services/SignalP-5.0/, accessed on 15 June 2025) was employed to anticipate and remove signal peptides from protein sequences, with the resulting sequences used in subsequent experiments [18].

### 2.2. In Silico Evaluation of Softshell Turtle Bone Collagen as a Precursor of Bioactive Peptides

According to the method of Zhang et al. [15], the “Profiles of Potential Biological Activity” tool in the BIOPEP-UWM database (https://biochemia.uwm.edu.pl/biopep-uwm/, accessed on 21 June 2025) was used to evaluate the biological activity of peptides released from specific sequences within type I collagen of soft-shelled turtle bone. To evaluate the feasibility of STBC as a bioactive peptide precursor, the occurrence frequency (*A*) of bioactive fragments within its protein sequence was determined by means of the formula presented below [19]:

(1) $$A=\frac{a}{N}$$

where *A* represented the frequency of occurrence of biologically active fragments in protein sequences, *a* represented the number of fragments possessing specific activity, and *N* represented the number of amino acid residues.

### 2.3. Simulated Enzymatic Hydrolysis and Preliminary Screening of Potential Functional Peptides

According to the method of Zhang et al. [15], the “Enzyme(s) Action” tool in the BIOPEP-UWM database (accessed on 27 June 2025) was used to simulate the enzymatic hydrolysis of the STBC sequence, thereby obtaining peptide fragments generated by different enzymes. Firstly, the sequences of soft-shelled turtle bone collagen were subjected to single-enzyme simulated hydrolysis using 28 enzymes available in the database, such as chymotrypsin A (EC: 3.4.21.1) and trypsin (EC: 3.4.21.4). The evaluation of enzymatic activity was grounded on the frequency of biologically active fragment occurrence and the degree of hydrolysis (*DH_T_*). The frequency of ACE-inhibitory fragments (*A*) was obtained grounded on the above Equation (1), while the theoretical degree of hydrolysis (*DH_T_*) was computed by Equation (2) [19]:

(2) $${\mathrm{DH}}_{T}=\frac{d}{D}\times 100\%$$

where *DH_T_* represented the theoretical degree of hydrolysis, *d* denoted the number of hydrolyzed peptide bonds within a protein or peptide chain, and *D* was the total number of peptide bonds present in a protein or peptide chain.

Subsequently, using A*_ACE inhibition_* as the primary evaluation index, several enzymes with the highest hydrolytic efficacy against the α1(I) and α2(I) chain sequences were selected. These selected enzymes were then employed in binary or ternary combinations to hydrolyze the STBC sequence, leading to the identification of the optimal enzyme cocktail. Following hydrolysis of the STBC with the identified optimal enzyme cocktail, a vast array of peptide sequences was obtained. Meanwhile, numerous studies have confirmed that the results of in silico virtual enzymatic hydrolysis of polypeptide sequences are highly consistent with those of actual in vitro enzymatic hydrolysis; thus, selecting the optimal multi-enzyme combination obtained via the aforementioned method is of equal practical significance [20,21]. To screen for peptides with potential bioactivity, these peptide sequences were aligned against established ACE-inhibitory peptides in the BIOPEP-UWM database (accessed on 3 August 2025) and those reported in existing scientific literature (searched 3 August 2025). Furthermore, the potentially novel ACE-inhibitory peptides with no prior reports in existing studies were uploaded to the ToxinPred (https://webs.iiitd.edu.in/raghava/toxinpred/index.html, accessed on 5 August 2025) and AllerCatPro 2.0 (https://allercatpro.bii.a-star.edu.sg, accessed on 5 August 2025) servers for the prediction of potential toxicity and allergenicity, respectively [22,23]. Ultimately, only those peptide fragments with predicted non-toxic and non-allergenic properties were selected for subsequent analysis.

### 2.4. Screening ACE-Inhibitory Peptides Based on Protein Language Models

To further specifically screen for potential ACE-inhibitory peptides, the web server pLM4ACE (https://sqzujiduce.us-east-1.awsapprunner.com, accessed on 7 August 2025) was employed. pLM4ACE is a protein language model (pLM) based on an embedded evolutionary scale modeling (ESM-2) architecture. Trained on extensive datasets, it serves as a web-based platform for screening potent ACE-inhibitory peptides. The server powering this website now offers access to three core classification models: logistic regression, support vector machine (SVM), and multilayer perceptron [24]. Subsequently, the candidate ACE-inhibitory peptide sequences were submitted to all three models. Those predicted as “high activity” by any of the models were combined to form the final set of selected peptides.

### 2.5. Molecular Docking Analysis of Screened Peptides with ACE

According to the method of Wu et al. [14], molecular docking of the screened ACE-inhibitory peptides was carried out using Discovery Studio 2019 (Dassault Systèmes Biovia, San Diego, CA, USA). For molecular docking assays, the crystal structure of human ACE (PDB ID: 1O86) was first obtained from the Protein Data Bank (https://www.rcsb.org/, accessed on 10 August 2025) and then subjected to a sequence of preprocessing operations through the “Prepare Protein” function in Discovery Studio. These steps comprised loop modeling, energy minimization, deletion of water molecules, removal of the bound ligand, and protonation. Based on the existing ligand lisinopril, the coordinates of the ACE ligand-binding site were determined as x: 41.232973, y: 33.971206, z: 46.544998 with a radius of 9 Å.

Additionally, Lisinopril (PubChem CID: 5362119) was selected as the reference ligand for molecular docking, as it is a canonical and highly effective ACE inhibitor. Its corresponding 3D molecular structure was acquired from the PubChem database. (https://pubchem.ncbi.nlm.nih.gov, accessed on 11 August 2025). Concurrently, the 3D structure of the screened peptide was built using the “Build and Edit Protein” tool in DS 2019. The resulting peptide structures were then prepared with the “Prepare Ligands” tool, which involved structural inspection and repair, protonation, charge assignment, and geometry optimization. Ultimately, energy minimization was performed on the molecular system via the “Minimization” tool.

Subsequently, semi-flexible molecular docking analysis was performed via the CDOCKER protocol in DS 2019. Specifically, the receptor was defined as rigid, while the ligands were processed as flexible. After extracting the ligands and performing redocking, the root-mean-square deviation (RMSD) between the ligand conformations after docking and those in the initial model was less than 2 Å. Taking the optimal binding conformations of each ligand-receptor complex as the research objects, the -CDOCKER Energy (-CE) and -CDOCKER Interaction Energy (-CIE) were calculated, which were used as the evaluation criteria for the docking results. Peptides exhibiting the highest -CIE and -CE values were identified as the most promising novel ACE inhibitory peptides and advanced to the subsequent screening stage [15]. Concurrently, a visual analysis was conducted to identify the interaction sites and types between the peptides and ACE, thereby elucidating the mechanism of ACE inhibition.

### 2.6. ADME and Physicochemical Property Prediction of ACE-Inhibitory Peptides

The physicochemical and pharmacokinetic ADME (absorption, distribution, metabolism, and excretion) properties of the peptides selected via molecular docking were evaluated in silico [15,25]. According to the method of Zhang et al. [15], the hydrophobicity, net charge, and isoelectric point of peptides were calculated with the ToxinPred server (https://webs.iiitd.edu.in/raghava/toxinpred/index.html, accessed on 20 August 2025). The sensory properties of the peptides were calculated utilizing the ‘Calculations’ tool within the “Sensory Peptides and Amino Acids” database (https://biochemia.uwm.edu.pl/biopep-uwm/, accessed on 20 August 2025) on the BIOPEP-UWM [26]. Additionally, the water solubility was evaluated via the SwissADME server (http://www.swissadme.ch, accessed on 22 August 2024) [27]. The ADME properties of the candidate peptides were evaluated in silico via the iDrug (https://drug.ai.tencent.com/en/, accessed on 22 August 2025), and their molecular weights were simultaneously calculated.

### 2.7. Screening of Hypertension-Related Potential Targets for the Selected Peptides

According to the method of Zhao et al. [28], the potential targets of the selected peptides were forecasted via the SwissTargetPrediction server (http://www.swisstargetprediction.ch, accessed on 25 August 2025) [29]. The SMILES formats of the peptides were input into the submission box of the SwissTargetPrediction server, with the species parameter set to “Homo sapiens” to predict potential targets. Notably, although numerous studies have applied SwissTargetPrediction for the prediction of small molecular peptides, this method still has inherent limitations. Specifically, the training dataset of SwissTargetPrediction is restricted to non-peptidic small molecular compounds, and its predictive model has thus not been optimized for peptide structures. This implies that the prediction results for peptides may not be as accurate as those for the authentic targets of small molecules [15,28,29]. Target genes associated with hypertension were acquired from GeneCards (https://www.genecards.org/, accessed on 1 September 2025) using the search term “Hypertension”. Overlapping targets between selected peptide potential targets and hypertension-related targets were plotted with an online Venn diagram tool. (https://www.bioinformatics.com.cn/static/others/jvenn/example.html, accessed on 1 September 2025), revealing the candidate targets for anti-hypertensive activity.

### 2.8. Protein-Protein Interaction (PPI) Network Analysis

The protein-protein interaction (PPI) network was established via the STRING database (http://string-db.org, accessed on 33 September 2025) using the overlapping targets of the identified peptides, which were implicated in anti-hypertensive properties as revealed by the Venn diagram analysis [30]. “*Homo sapiens*” was designated as the target organism, with the minimum interaction score set to the highest confidence (>0.9) [28]. The constructed PPI network was then imported into Cytoscape 3.10.0 for visualization and further analysis. Subsequently, the CytoHubba plugin was used to identify the top 10 significant targets via eight topological analysis methods. Finally, the hub targets were determined by taking the intersection of the results from all eight algorithms [31].

### 2.9. GO and KEGG Pathway Enrichment Analysis

According to the method of Cheng et al. [31], enrichment analyses of Gene Ontology (GO) terms and Kyoto Encyclopedia of Genes and Genomes (KEGG) pathways were conducted with the use of the DAVID database. (https://davidbioinformatics.nih.gov/home.jsp, accessed on 10 September 2025). The identified potential anti-hypertensive targets were submitted to the DAVID platform with the species specified as “Homo sapiens” and a significance threshold of *p* < 0.01. The filtered results were sorted and saved according to the number of targets involved in each term, thereby facilitating the identification of key biological processes and pathways. The screening results were visualized via an online tool (https://www.bioinformatics.com.cn/, accessed on 12 September 2025), where bar plots and bubble charts were generated for analytical purposes [28,32].

### 2.10. Statistical Analysis

All figures and charts in this study were plotted using Origin V2021 software (OriginLab Corporation, Northampton, NC, USA) and Microsoft PowerPoint 2016, while all tables were constructed with Microsoft Excel 2016. Hierarchical cluster analysis (HCA) was performed via the plug-in application of Origin V2021.

## 3. Results and Discussion

### 3.1. Evaluation of the Potential of STBC as a Precursor of Bioactive Peptides

**Figure 1:**
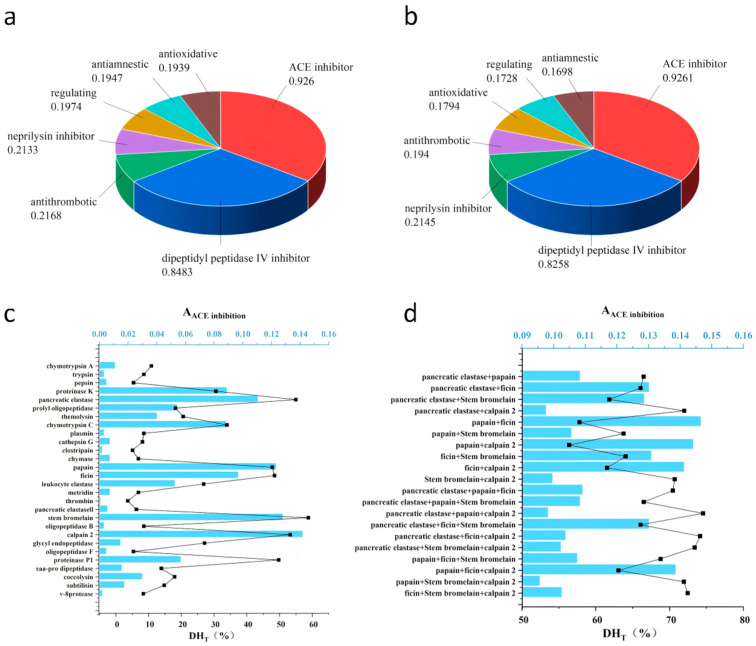
(**a**,**b**) Computer-aided bioactivity evaluation of peptides released from α1 (I)-chain and α2 (I)-chain of STBC. (**c**,**d**) ACE inhibitory frequency and theoretical degree of hydrolysis (DH_t_) of α1 and α2 chains from STBC after simulated proteolysis. STBC, softshell turtle bones collagen.

The potential of softshell turtle bone collagen (STBC) to generate bioactive peptides was evaluated through computer simulation. Figure 1a,b display the frequency of bioactive fragments with specific activities for the α1(I) and α2(I) chains, respectively. The findings revealed that ACE-inhibitory peptides showed the highest occurrence frequency in both the α1(I) and α2(I) chains of STBC (A = 0.8947 and A = 0.9261, respectively), which demonstrates that STBC has substantial potential to serve as a precursor for ACE-inhibitory peptides. It is noteworthy that DPP-IV inhibitory activity also exhibited a high frequency of occurrence in the α1(I) and α2(I) chains of STBC (A = 0.8483 and A = 0.8258, respectively), indicating their additional potential as a precursor for DPP-IV-inhibitory peptides.

### 3.2. Simulated Proteolysis of STBC

Enzymatic hydrolysis of proteins is a crucial process for generating bioactive peptides with physiological functions such as ACE inhibition, antioxidant, and anti-diabetic activities. Nevertheless, conventional methods, which combine enzymatic hydrolysis with purification and identification, are often time-consuming and labor-intensive. In contrast, in silico proteolysis offers an efficient strategy to predict enzyme-specific cleavage sites and potential bioactive fragments directly from known protein sequences, thereby serving as a valuable pre-screening tool in peptide discovery [33,34]. This study employed 28 enzymes from the BIOPEP-UWM database for simulated proteolysis. The performance of each enzyme was evaluated using the frequency of bioactive fragments (A) and the degree of hydrolysis (DH) as key metrics. The BIOPEP-UWM database has emerged as a vital tool for studying bioactive peptides in recent years, particularly for investigating peptide substances derived from food that constitute dietary components for chronic disease prevention. Currently, the database contains 5490 bioactive peptide sequences associated with multiple types of biological functions, with 1235 peptide fragments showing the capacity to inhibit ACE activity [26,28].

Given the composition of type I collagen (two α1(I) chains and one α2(I) chain), the overall *A* and *DH_T_* for STBC were calculated as weighted averages based on this 2:1 ratio. As shown in Figure 1c, the hydrolysate generated by calpain 2 (EC 3.4.22.53) demonstrated the most potent ACE inhibitory activity (*A* = 0.1419). However, it is noteworthy that despite having the highest *A*, this enzyme did not yield the highest *DH_T_*. Collectively, the results from the 28 enzymes indicate that the ACE-inhibitory activity of STBC hydrolysates is not strongly positively correlated with the degree of hydrolysis under different enzymatic conditions. This result corresponds to the research outcomes of Zhang et al. [35], who likewise reported no statistically significant correlation (*p* > 0.05) between ACE-inhibitory activity and the degree of hydrolysis (*DH*) in three legume species under different enzymatic hydrolysis conditions. Based on the enzymatic hydrolysis efficiency, five enzymes were selected for subsequent combinatorial enzymatic hydrolysis studies: pancreatic elastase (EC: 3.4.21.36), papain (EC: 3.4.22.2), ficin (EC: 3.4.22.3), stem bromelain (EC: 3.4.22.32), and calpain 2 (EC: 3.4.22.53).

Subsequently, to identify more suitable enzymatic conditions, STBC was subjected to simulated hydrolysis using enzyme combinations, aiming to enhance both the A*_ACE inhibition_* and *DH_T_*. As shown in Figure 1d, the multi-enzyme hydrolysis exhibited significantly enhanced ACE inhibitory activity and *DH_T_* compared to single-enzyme treatments. Among these combinations, the papain (EC 3.4.22.2) and ficin (EC 3.4.22.3) mixture yielded the highest ACE inhibitory activity (*A* = 0.1464). Nevertheless, consistent with our previous findings from single-enzyme hydrolysis, the multi-enzyme combination demonstrating the highest ACE inhibitory activity failed to achieve the maximum *DH_T_*. It is noteworthy that although the combination of multiple enzymes significantly increased *DH_T_* levels compared with single-enzyme treatment, this enhancement was not accompanied by a significant elevation in bioactivity. This phenomenon may be ascribed to excessive hydrolysis during combinatorial enzymatic treatment, which potentially generates increased amounts of free amino acids and converts potential bioactive peptides into shorter, inactive fragments [14]. Consequently, despite the elevated *DH_T_*, no substantial enhancement in ACE inhibition was observed, further validating the lack of significant correlation between these parameters across different enzymatic hydrolysis conditions [35]. Therefore, further research will proceed with the hydrolysate obtained using the papain (EC 3.4.22.2) and ficin (EC 3.4.22.3) combination.

### 3.3. Primary Screening of Peptide Fragments Released from STBC via Simulated Enzymatic Hydrolysis

**Figure 2:**
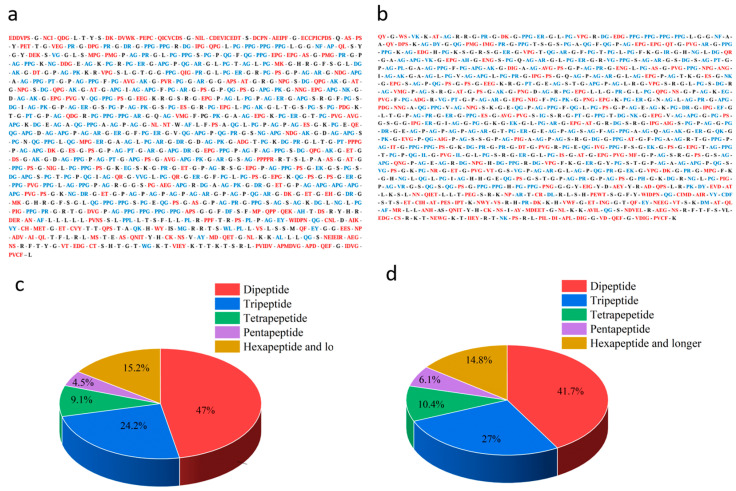
(**a**,**b**) Results of peptide release from simulated hydrolysis of α1(I)-chain and α2(I)-chain of STBC under optimal enzymatic hydrolysis combination (papain-ficin). Known ACE inhibitory peptides obtained from BIOPEP-UWM database or previous peer-reviewed reports are indicated in blue, while peptide sequences not reported to have ACE inhibitory activity are marked in red. (**c**,**d**) Length distribution of peptide sequences released from α1(I)-chain and α2(I)-chain of STBC via simulated hydrolysis under optimal enzymatic hydrolysis combination (papain-ficin). STBC, softshell turtle bones collagen.

Figure 2a,c illustrate the release profile and length distribution, respectively, of peptide fragments derived from the α1(I) chain of STBC through simulated hydrolysis using the papain + ficin enzyme combination. The simulated enzymatic hydrolysis of the α1(I) chain yielded 66 distinct peptide fragments (Figure 2a), consisting of 31 dipeptides, 16 tripeptides, 6 tetrapeptides, 3 pentapeptides, and 10 peptides containing six or more amino acid residues (Figure 2c). A total of 24 dipeptides and tripeptides among the released peptides were verified to exhibit ACE-inhibitory activity according to the BIOPEP-UWM database, including: PR, AY, AF, AP, VG, AG, MG, WG, QG, AI, EG, NG, PG, AR, PT, AH, EK, DF, WL, PPL, ER, DR, AK, WY. In addition, three other peptide sequences have been reported to exhibit ACE inhibitory activity: PPG (23.14% inhibition rate at a concentration of 1000 μL/mL), APG (activity confirmed with no specified parameters), and PK (IC_50_ = 4092 μM) [36,37,38].

Simultaneously, simulated hydrolysis of the α2(I) chain yielded 115 distinct peptide fragments (Figure 2b), comprising 48 dipeptides, 31 tripeptides, 12 tetrapeptides, 7 pentapeptides, and 17 peptides with six or more amino acid residues (Figure 2d). Among these peptides, 35 dipeptides and tripeptides with confirmed ACE inhibitory activity were identified in the BIOPEP-UWM database, specifically: IR, MF, VY, PR, AY, PL, VK, AF, VG, IG, AG, QG, EG, NG, PG, VR, QK, DG, NF, NK, AR, EY, PT, AH, EK, PH, DY, DM, IL, EF, ER, DR, DL, AK, DL. Five additional peptide sequences from the remaining pool were documented in previous studies for ACE inhibition: PPG (23.14% inhibition at 1000 μL/mL), APG, PK (IC_50_ = 4092 μM), VY (IC_50_ = 5.2 μM), and CDF (IC_50_ = 192.17 ± 2.46 μM) [36,37,38,39,40].

Following integration of the peptide release results from STBC’s α1(I) and α2(I) chains, all peptides reported in previous studies or present in the BIOPEP-UWM database were excluded from further analysis. Subsequently, the toxicity of the screened peptides was predicted with the aid of ToxinPred. The results demonstrated that only one long peptide sequence, ECCPICPDS, exhibited toxicity. Concurrently, allergenicity prediction for the simulated enzymatic hydrolysate peptides was conducted using the online tool AllerCatPro 2.0. The results demonstrated that, similar to the toxicity prediction, only one longer peptide sequence, CDEVICEDT, was predicted to be allergenic. Ultimately, following exclusion of the two peptides with toxic and allergenic properties, 105 peptides with potential ACE inhibitory activity were selected from STBC for subsequent screening.

### 3.4. Screening of ACE-Inhibitory Peptides by Large Data Language Models

The potential ACE inhibitory peptides obtained from STBC via simulated hydrolysis were evaluated using the pLM4ACE protein language model. Developed by Du et al. [24], based on the ESM-2 algorithm, the model applies deep learning embeddings for peptide feature encoding and combines Logistic Regression, SVM, and MLP classifiers to create an integrated prediction system. Compared to conventional models based on physicochemical properties or amino acid composition, pLM4ACE demonstrates significantly enhanced predictive accuracy for ACE-inhibitory peptides. The optimal model configuration (Logistic Regression with ESM-2 embedding) achieved a balanced accuracy of 0.883 and an AUC value of 0.96 on an independent test set [41].

In this study, the 105 softshell turtle bone collagen peptide sequences obtained from previous simulated enzymatic hydrolysis were subjected to prediction using the three classification models in pLM4ACE. According to the screening criteria of pLM4ACE, a peptide was designated as a potential ACE-inhibitory peptide if any of the models (Logistic Regression, SVM, or MLP) predicted ACE-inhibitory activity. As presented in Table 1, the screening process identified 27 peptides predicted to possess ACE-inhibitory activity, accounting for 25.7% of the total peptides. This result indicated that even after excluding the peptides previously identified to possess ACE inhibitory activity in Section 3.3, STBC still contains a high proportion of peptides that may possess ACE inhibitory activity, reaffirming its promise as a precursor of natural anti-hypertensive peptides and corroborating the findings presented in Section 3.1.

Furthermore, subsequent analysis revealed that all peptides predicted to be active exhibited short sequence lengths (2–7 amino acids), with the majority containing hydrophobic residues. These properties are consistent with the extensively documented characteristics of typical ACE-inhibitory peptides reported in relevant literature [14]. Extensive research has confirmed a strong association between the structural characteristics of peptides and their biological activities. Specifically, short peptides composed of 2–10 amino acid residues are more likely to function as potent ACE inhibitors [6]. Moreover, the inhibitory potential of peptides is strongly correlated with their amino acid sequences, with the content of hydrophobic residues being a particularly critical factor. This phenomenon is primarily attributed to the ability of hydrophobic side chains to enhance interactions with the functional hydrophobic pockets of ACE, thereby strengthening inhibitory effects [42].

### 3.5. Molecular Docking Analysis

Molecular docking technology holds significant importance in the virtual enzymatic hydrolysis screening of functional peptides. It enables rapid evaluation of the binding ability with target proteins (such as ACE) among numerous candidate peptides, thereby screening out those with potentially high activity and significantly reducing the time and cost of experimental screening. Meanwhile, analysis of hydrogen bonds, hydrophobic interactions, and charge interactions within the docking complexes enables the identification of key binding sites and action mechanisms between peptides and target proteins, thereby laying a theoretical basis for subsequent in vitro validation and structural optimization. Therefore, molecular docking has become one of the important means for the computational screening of functional peptides [43,44].

Table 1 presents the -CDOCKER energy (-CE) and -CDOCKER interaction energy (-CIE) between the peptides and ACE. In semi-flexible docking, -CIE corresponds to the minimal binding energy of the ligand-receptor complex, whereas -CE comprises both the binding energy and the internal strain energy of the ligand. Highly positive values of -CIE or -CE generally correlate with substantial energy release during interaction, indicating strong binding affinity of the ligand-receptor complex. Consequently, both parameters have been extensively employed as key criteria for screening target-specific ligands or receptors in previous studies [15,45,46]. The results demonstrated that all 27 candidate peptides formed stable binding conformations with ACE (-CIE, -CE > 0). Notably, the binding energies of four of these peptides are higher than that of lisinopril, indicating that they may exhibit favorable binding affinity and potential ACE inhibitory activity.

**Figure 3:**
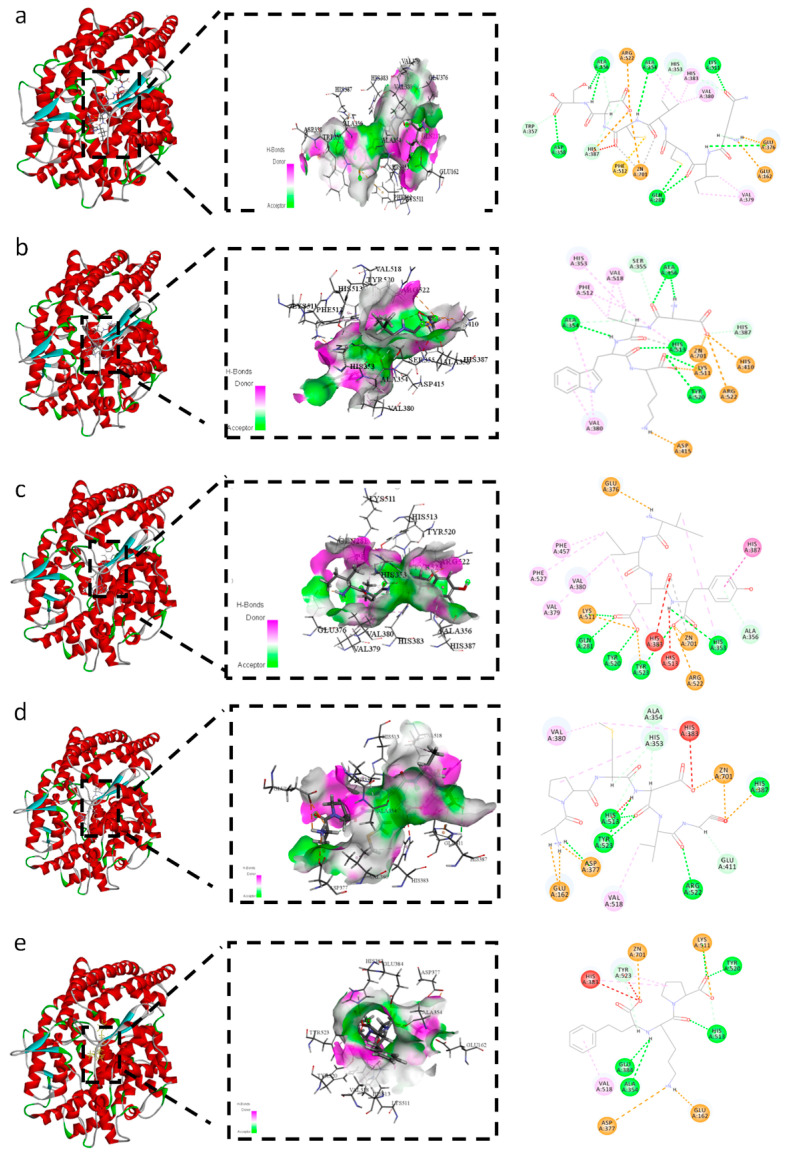
3D and 2D diagrams of the binding of screened peptides and lisinopril to the ace active site. (**a**) QICVCDS; (**b**) DVWK; (**c**) IIEY; (**d**) APMDVG; (**e**) Lisinopril.

**Figure 4:**
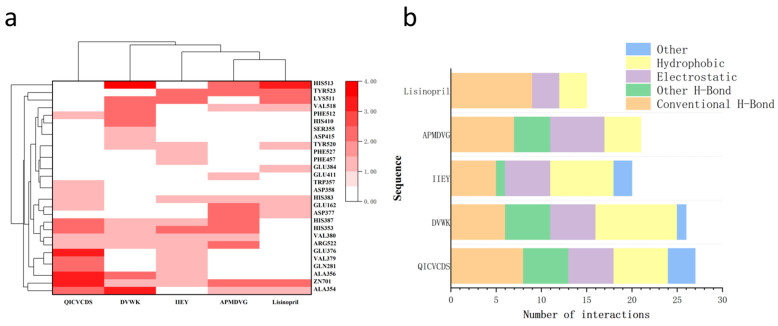
(**a**) HCA analysis of non-bonded interaction sites between screened peptides, lisinopril, and ACE. The color gradient indicates the number of non-bonding interactions with specific amino acid residues or Zn^2+^. (**b**) Statistics of non-bonded interaction patterns for interactions between screened peptides, lisinopril, and ACE.

Figure 3a–e respectively depict the non-bonded interactions of the four high-binding-energy peptides and Lisinopril with the ACE receptor, illustrated in both 3D and 2D diagrams. The active site of ACE is characterized by three constituent binding pockets: S1 (Ala354, Glu384, and Tyr523), S2 (Gln281, His353, Lys511, His513, and Tyr520), and S1’ (Glu162) [47]. Moreover, a statistical analysis of the non-bonded interaction sites and modes was performed, with the corresponding results presented in Figure 4a and Figure 4b, respectively. The binding of Lisinopril to ACE is mainly mediated by hydrogen bonds, including five conventional hydrogen bonds and four carbon-hydrogen bonds, followed by three electrostatic interactions and three hydrophobic interactions (Figure 4). All four candidate peptides (QICVCDS, DVWK, IIEY, APMDVG) formed stable complexes with ACE through hydrogen bonding, electrostatic, and hydrophobic interactions, albeit with distinct interaction patterns. QICVCDS exhibited the maximum number of hydrogen bonds and electrostatic interactions, indicating a predominantly polar-driven binding mode that enables highly directional anchoring within the catalytic active site. In contrast, DVWK exhibited the most extensive hydrophobic interactions, consistent with stabilization primarily through embedding into the S1/S2 pockets. APMDVG displayed a balanced distribution among the three interaction types, while IIEY, despite forming fewer hydrogen bonds, achieved specific contacts with key residues via electrostatic and hydrophobic forces. All four peptides demonstrated more complex multi-point binding patterns compared to the reference ligand Lisinopril, engaging crucial residues including Ala354, Tyr523, Gln281, and His353, thereby physically obstructing substrate access to ACE. Meanwhile, the presence of ZN701 was observed in the docking results of the four peptides and lisinopril with ACE. As shown in Figure 4a, the number of Zn^2+^ participating in binding in Lisinopril was 2, consistent with the count for APMDVG. DVWK and IIEY exhibited the lowest number at 1, while QICVCDS showed the highest number, reaching 3. ZN701 interacts via attractive charge–metal receptor interactions, and the interaction between the ligand and Zn^2+^ in ACE positively contributes to the enzyme’s inhibitory capacity [48].

Overall, the candidate peptides stabilized their complexes via synergistic non-bonded interactions. In comparison, although Lisinopril formed numerous hydrogen bonds, its limited hydrophobic and electrostatic contributions resulted in lower docking scores than the four peptides, underscoring the latter’s considerable potential as ACE inhibitor. Wu et al. [14] also reported that the synergistic effect of multiple hydrogen bonds, attractive electrostatic interactions and hydrophobic interactions can enhance the inhibitory activity of peptides This is specifically evidenced by a higher -CIE value, which corresponds to a more favorable binding capacity.

### 3.6. Physicochemical and Pharmacokinetic Properties of Peptide Candidates

The physicochemical properties and absorption characteristics of bioactive peptides are recognized as crucial factors influencing their biological activity [28]. Therefore, this study systematically characterized the physicochemical parameters and pharmacokinetic (ADME) profiles of four novel ACE inhibitory peptide candidates through in silico analysis, with detailed results summarized in Table 2.

Previous studies have indicated that ACE-inhibitory peptides are typically short-chain peptides (approximately 2–12 amino acids) with molecular weights generally below 3 kDa. Notably, low-molecular-weight peptides (<1 kDa) often demonstrate enhanced ACE inhibitory activity [49]. The molecular weights of the four peptides obtained in this study were determined to be between 536.6 and 766.9 Da, with peptide lengths of 4–7 residues. Furthermore, these candidate peptides exhibited relatively high hydrophobicity (>8.5 kcal/mol), which represents a typical characteristic of ACE inhibitory peptides. This property enhances their binding to the hydrophobic pockets of ACE, thereby potentially strengthening binding affinity [43]. With the exception of DVWK, the remaining three peptides exhibited isoelectric points (pI) ranging from 3.05 to 3.14, all significantly below 7. This acidic nature confers a net negative charge (1–2 units) in neutral solvents, which may facilitate electrostatic interactions with positively charged residues in the ACE protein. DVWK exhibited a near-neutral isoelectric point (pI = 6.77), resulting in an almost neutral net charge (0) under physiological conditions. The peptide demonstrated weak overall polarity but strong hydrophobicity (11.79 kcal·mol^−1^). This set of characteristics determines that its interaction with the ACE catalytic center primarily relies on hydrophobic embedding rather than electrostatic attraction, consistent with our molecular docking results showing the highest number of hydrophobic interactions (9 sites) and relatively few electrostatic contacts. The aqueous solubility (logS) of all candidate peptides ranged from –0.41 to 1.51, collectively indicating favorable solubility properties. This characteristic suggests promising potential for further pharmaceutical applicability [17]. Sensory profiling revealed that the peptides generally exhibited bitter or umami characteristics, aligning with the documented sensory attributes of most reported ACE-inhibitory peptides [49].

On the other hand, the four candidate peptides exhibited promising ADME characteristics, as detailed in Table 2. All four candidate peptides demonstrated high human intestinal absorption (HIA) probabilities exceeding 0.4, with QICVCDS and IIEY achieving remarkable values of 0.99 and 1.00, respectively, indicating excellent absorption potential in humans. However, among the four peptides, only DVWK exhibited a human oral bioavailability (HOB) value approaching 20 (actual measurement: 18). This suggests that encapsulation or other targeted delivery strategies may be necessary to enhance its bioavailability [15]. Although HIA and HOB values predicted by SwissADME do not directly reflect gastrointestinal stability, peptides with higher predicted HIA and HOB values are more likely to exert systemic bioactivities if they survive gastrointestinal digestion. Among the four peptides, DVWK exhibited the highest HOB value, suggesting a relatively greater probability of remaining intact during gastrointestinal transit. In contrast, peptides with extremely high HIA values but lower HOB values, such as QICVCDS and IIEY, may possess strong intestinal absorption potential but still be limited by enzymatic degradation and first-pass metabolism. These results indicate that gastrointestinal stability may vary among the peptides and warrant further verification by in vitro digestion models. Furthermore, all candidate peptides demonstrated low CYP450 inhibition probabilities (<0.25), indicating that none were predicted to act as substrates or inhibitors of CYP450 isoforms. This suggests a favorable safety profile with minimal risk of human metabolic interactions [50].

### 3.7. Potential Antihypertensive Targets of Candidate Peptides and PPI Network Analysis

**Figure 5:**
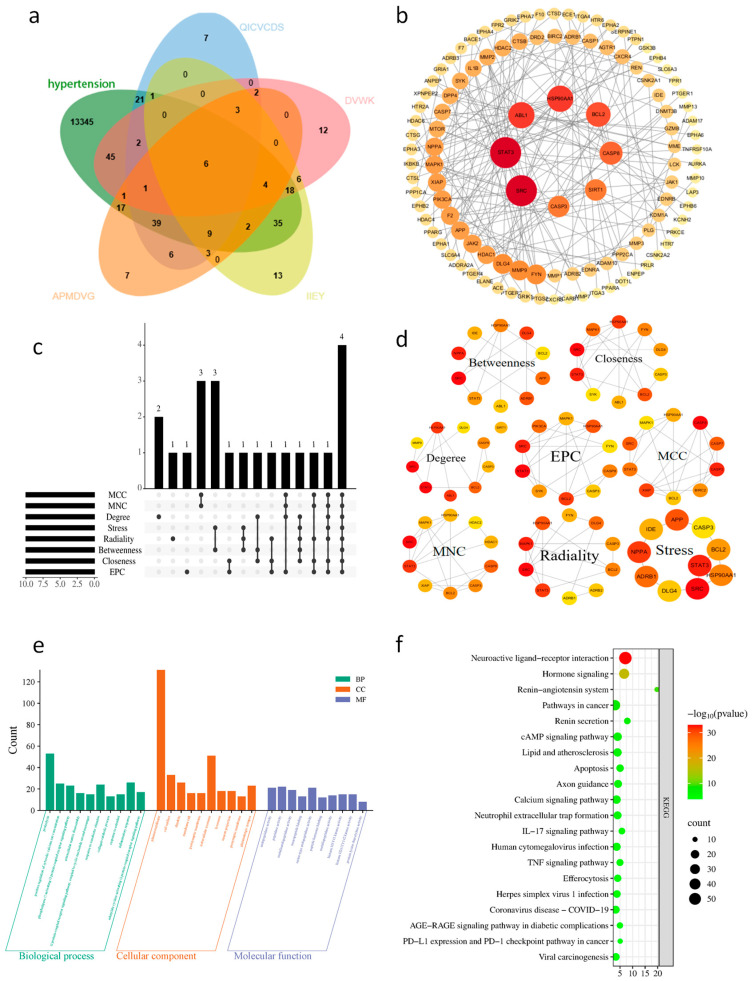
(**a**) Venn diagram of overlapping targets between identified peptides and antihypertensive effects. (**b**) PPI network diagram of antihypertensive targets for identified peptides. (**c**,**d**) Intersection diagram and network diagram of the top 10 key antihypertensive targets obtained via eight topological analysis algorithms (**e**,**f**) Go functional enrichment and KEGG enrichment pathways of antihypertensive targets for identified peptides.

Hypertension is a chronic condition characterized by polygenic and multi-pathway involvement, encompassing intricate networks of vasoconstriction/vasodilation regulation, inflammatory responses, oxidative stress, and the renin-angiotensin system. Given this complexity, single-target strategies often prove inadequate for comprehensively intervening in its pathological process. Network pharmacology has emerged as a powerful way for elucidating multi-target mechanisms in complex diseases by integrating “active component-target-disease” relationships and constructing protein-protein interaction (PPI) networks [51]. Zhai et al. [52] demonstrated through network pharmacology and PPI analysis that *Pinellia ternata* exerts anti-hypertensive effects by targeting proteins such as AKT1, VEGFA, and JUN. Consequently, this research adopted network pharmacology to predict the anti-hypertensive targets and protein interaction networks of the ultimately identified novel peptide compounds, with the results presented in Figure 5a and Figure 5b, respectively.

Employing the SwissTargetPrediction tool, we identified 100 potential targets for each of the four candidate peptides. Simultaneously, 13,345 hypertension-related targets were obtained through systematic screening of the GeneCards database. Subsequently, Venn diagram analysis was performed to compare these 400 peptide-associated targets with the 13,345 hypertension-related targets, ultimately identifying 201 overlapping targets as potential anti-hypertensive targets for the candidate peptides (Figure 5a).

The 201 intersecting targets mentioned above were analyzed in depth via the String database, and a PPI network containing 112 nodes (excluding disconnected nodes) and 184 connecting edges was constructed (Figure 5b). The intricate network was subsequently analyzed via the CytoHubba plugin in Cytoscape. Specifically, nodes were ranked according to eight topological analysis algorithms: MCC, MNC, EPC, Degree, Closeness, Betweenness, Radiality, and Stress. The results are illustrated in Figure 5c,d. Based on the scores generated by the algorithm, the genes were sorted in descending order, and the top 10 were defined as hub genes. (Table 3). Four targets, namely SRC (src proto-oncogene), HSP90AA1 (heat shock protein 90 alpha family class A member 1), BCL2 (BCL2 apoptosis regulator), and STAT3 (signal transducer and activator of transcription 3), were found to overlap in all algorithms and were identified as the hub genes of the four antihypertensive peptides identified (Figure 5d).

### 3.8. GO Analysis and KEGG Enrichment Analysis of Target Genes

To further investigate the multiple mechanisms underlying the defensive effects of candidate peptides against hypertension, GO annotation and KEGG enrichment analysis were carried out on the 201 predicted targets via the DAVID. Gene Ontology (GO) analysis generally encompasses three core dimensions for functional annotation: biological process (BP), molecular function (MF), and cellular component (CC). Figure 5e shows the top 10 terms with the largest number of targets and *p* < 0.01 in the BP, CC, and MF categories of the GO analysis. As revealed by the BP analysis, these targets are predominantly engaged in proteolysis, positive regulation of cytosolic calcium ion concentration, phospholipase C-activating G protein-coupled receptor signaling pathway, extracellular matrix disassembly, and other related processes. CC analysis indicated that these targets are primarily localized to the plasma membrane, cell surface, dendrites, and membrane rafts, among other components. The plasma membrane showed a significantly higher count value (131) compared to other cellular locations. Regarding MF, these targets are primarily involved in endopeptidase activity, peptidase activity, metalloendopeptidase activity, neuropeptide binding, and related processes.

Furthermore, KEGG pathway enrichment analysis indicated that the identified targets exhibited significant enrichment in multiple pathways. The top 20 pathways with the highest enrichment factors (*p* < 0.01) are depicted in Figure 5f. The results demonstrated that these targets were primarily enriched in pathways including the renin-angiotensin system, neuroactive ligand-receptor interaction, hormone signaling, and renin secretion. It is noteworthy that the renin-angiotensin system (RAS) was the most significantly enriched pathway (enrichment factor = 19.74), suggesting that the peptides may exert antihypertensive effects by modulating RAS. This finding is consistent with the perspective put forward by Majumder et al. [53] in their review on antihypertensive peptide targets. However, the inference of therapeutic mechanisms via network pharmacology has certain limitations; future studies should establish cellular or animal models to further investigate and validate the biological action pathways of these ACE inhibitory peptides.

## 4. Conclusions

This study systematically identified novel peptides with potential angiotensin-converting enzyme (ACE)-inhibitory activity from softshell turtle (*Pelodiscus sinensis*) bone collagen (STBC) using an integrated in silico strategy. Bioinformatics analysis confirmed that STBC (α1(I) chain: K7FHL1; α2(I) chain: K7G8R1) is a high-quality precursor of ACE-inhibitory peptides, with the frequency of ACE-inhibitory fragments reaching 0.8947 and 0.9261 in the two chains, respectively. The papain-ficin combination was screened as the optimal enzymatic system for STBC hydrolysis. Following toxicity/sensitization prediction, pLM4ACE activity evaluation, and molecular docking validation against ACE (PDB ID: 1O86), four novel peptides (QICVCDS, DVWK, IIEY, APMDVG) were identified to exhibit high binding affinity. Inferred via web server, these peptides (molecular weight: 536.6–766.9 Da) exhibited favorable physicochemical properties (e.g., appropriate hydrophobicity, good water solubility) and ADMET profiles (high human intestinal absorption, low metabolic risk), meeting the basic requirements for bioactive peptide development. Network pharmacology further revealed that the candidate peptides might exert antihypertensive effects by targeting SRC, HSP90AA1, BCL2, and STAT3, and regulating the renin–angiotensin system and neuroactive ligand–receptor interaction pathways. However, the above findings are only preliminary, and further in vitro/in vivo validation is still required. Overall, this study not only provides an economical and efficient in silico approach for screening food-derived ACE-inhibitory peptides but also lays a theoretical foundation for the high-value utilization of softshell turtle aquatic resources and the development of natural antihypertensive functional ingredients. Notably, this method still has limitations and can only be used as a preliminary screening tool, rather than a direct basis for determining the biological activity of peptides.

## Figures and Tables

**Table 1:** Polypeptides screened via the pLM4ACE server and their molecular docking results.

Sequence	LR	SVM	MLP	-CE (kcal/ mol)	-CIE (kcal/ mol)
QICVCDS	high activity	high activity	high activity	125.954	118.496
DVWK	high activity	low & non-activity	low & non-activity	117.488	106.022
IIEY	high activity	high activity	high activity	105.96	105.308
APMDVG	high activity	low & non-activity	low & non-activity	100.198	104.216
VIEY	high activity	high activity	high activity	107.679	101.273
CR	high activity	high activity	high activity	51.7598	98.9681
DCPN	high activity	high activity	high activity	84.6444	95.1822
AVIL	low & non-activity	high activity	low & non-activity	97.2242	91.1243
NWY	high activity	high activity	high activity	94.1639	90.5661
CIMD	high activity	high activity	high activity	87.2086	87.2189
CDF	high activity	high activity	high activity	89.6301	85.0382
VWF	high activity	high activity	high activity	84.9517	82.9004
DPG	high activity	low & non-activity	low & non-activity	82.186	80.9088
CNL	high activity	high activity	high activity	88.73	80.173
IPT	high activity	high activity	high activity	60.7788	78.4556
CVY	high activity	high activity	high activity	83.7145	77.1025
ANH	low & non-activity	high activity	high activity	90.0769	76.6198
WY	high activity	high activity	high activity	74.7127	75.5368
CIH	high activity	high activity	high activity	76.0867	74.1174
APL	low & non-activity	high activity	high activity	62.8983	72.4292
APD	high activity	low & non-activity	high activity	51.6015	72.3056
PPF	low & non-activity	high activity	high activity	47.0456	71.9965
VL	high activity	high activity	high activity	70.8135	66.6751
VY	high activity	high activity	high activity	71.7841	66.6652
CH	high activity	high activity	high activity	70.8717	66.3302
CS	high activity	low & non-activity	high activity	65.5785	60.116
MP	high activity	high activity	high activity	38.9863	55.042
Lisinopril (positive control)				93.7579	103.348

LR, Logistic Regression; SVM, Support Vector Machine; MLP, Multi-Layer Perceptron; -CE, -CDOCKER energy; -CIE, -CDOCKER interaction energy.

**Table 2:** Physicochemical and pharmacokinetic ADME properties of novel ACE inhibitory peptide candidates from STBC through in silico analysis.

Peptide	QICVCDS	DVWK	IIEY	APMDVG
Molecular Weight	766.9	546.63	536.63	588.68
Isoelectric point (pI)	3.05	6.77	3.14	3.13
Net charge	−1	0	−1	−1
Hydrophobicity/ (Kcal/mol)	11.15	11.79	8.58	12.2
Sensory quality	bitter, salty, sour, sweet, umami	astringent, bitter, bitterness suppressing, salty, sour, sweet, umami	bitter, sour, umami	bitter, salty, sour, sweet, umami
Water solubility (log *S*)	0.83	1.51	−0.41	1.25
HIA probability	0.99	0.67	1	0.42
HOB (%)	9	18	14	13
P-gp substrate probability	0.46	0.74	0.85	0.42
P-gp inhibition probability	0.93	0.85	0.88	0.8
CYP450 substrate probability	0.16	0.07	0.22	0.13
CYP1A2 inhibition probability	0.27	0.37	0.51	0.27
CYP2C19 inhibition probability	0.22	0.21	0.36	0.2
CYP2C9 inhibition probability	0.15	0.14	0.4	0.13
CYP2D6 inhibition probability	0.15	0.23	0.33	0.19
CYP3A4 inhibition probability	0.16	0.28	0.33	0.24

pI, isoelectric point; log *S*, the logarithmic value of solubility (M) in water; HIA, human intestinal absorption; HOB, human oral bioavailability; P-gp, P-glycoprotein; CYP450, cytochrome P450; CYP1A2, CYP2C19, CYP2C9, CYP2D6, and CYP3A4, isoforms of cytochrome P450; STBC, softshell turtle bones collagen.; ADME, absorption, distribution, metabolism, and excretion.

**Table 3:** The top 10 hub genes of antihypertensive targets for identified peptides ranked by CytoHubba.

Catalog	Rank Methods in CytoHubba							
	MCC	MNC	Deg	Str	Rad	Bet	Clo	EPC
Gene symbol	CASP8	CASP8	SRC	SRC	SRC	SRC	SRC	SRC
top10	HSP	HSP	STAT3	STAT3	STAT3	STAT3	STAT3	STAT3
	BCL2	BCL2	SIRT1	IDE	FYN	IDE	FYN	FYN
	MAPK1	MAPK1	ABL1	ADRB1	ADRB2	ABL1	SYK	SYK
	XIAP	XIAP	MMP9	DLG4	ADRB1	ADRB1	ABL1	PIK3CA
	CASP3	CASP3	CASP3	CASP3	CASP3	DLG4	CASP3	CASP3
	CASP7	CASP7	DLG4	NPPA	DLG4	NPPA	DLG4	MAPK1
	STAT3	STAT3	BCL2	HSP	MAPK1	BCL2	MAPK1	HSP
	SRC	SRC	HSP	BCL2	BCL2	HSP	BCL2	BCL2
	BIRC2	BIRC2	CASP8	APP	HSP	APP	HSP	CASP8

Deg, Degree; Str, Stress; Rad, Radiality; Bet, Betweenness; Clo, Closeness; HSP, HSP90AA1.

## Data Availability

The original contributions presented in this study are included in the article. Further inquiries can be directed to the corresponding authors.
